# Top‐down effects of filter‐feeding fish and bivalves moderate bottom‐up effects of nutrients on phytoplankton in subtropical shallow lakes: An outdoor mesocosm study

**DOI:** 10.1002/ece3.10567

**Published:** 2023-09-25

**Authors:** You Zhang, Ruijie Shen, Kuanyi Li, Qisheng Li, Huihui Chen, Hu He, Xiaohong Gu, Zhigang Mao, Richard K. Johnson

**Affiliations:** ^1^ State Key Laboratory of Lake Science and Environment, Nanjing Institute of Geography and Limnology Chinese Academy of Sciences Nanjing China; ^2^ Department of Aquatic Sciences and Assessment Swedish University of Agricultural Sciences Uppsala Sweden; ^3^ Sino‐Danish College University of Chinese Academy of Sciences Beijing China; ^4^ University of Chinese Academy of Sciences Beijing China; ^5^ Huaiyin Normal University Huaiyin China

**Keywords:** algae control, biomanipulation, eutrophication, food web, lake restoration

## Abstract

Biomanipulation has been widely used in the ecological restoration of eutrophic lakes for decades. However, biomanipulation is prone to failure if external nutrient loads are not reduced. In order to explore the importance of filter‐feeding fish and bivalves on algal control, an outdoor mesocosm experiment was conducted using different nutrient concentrations. Four treatments simulating daily loads of nutrients in Lake Taihu were studied: current, two times, and three times average daily loads of nutrients with both fish (*Aristichthys nobilis*) and Asian clam (*Corbicula fluminea*) and as a control current daily loads without fish or bivalves. Results showed that stocking of filter‐feeding fish and bivalves (80 g m^−3^ bighead carp; 200 g cm^−2^ clams) at two times daily nutrient loads could effectively control water column Chl *a* concentrations and phytoplankton biomass. At higher nutrient concentrations (TN ≥ 260 μg L^−1^ d^−1^; TP ≥ 10 μg L^−1^ d^−1^), top‐down control of filter‐feeding fish and bivalves was less effective and bottom‐up effects resulted in significant increases of Chl *a* concentration. Thus, as phytoplankton biomass in freshwater ecosystems is determined by both the top‐down effects of predators and the bottom‐up effects of nutrients, external loadings should be controlled when filter‐feeding fish and bivalves are used for algal control to ensure the efficacy of biomanipulation.

## Graphical abstract

1. A combination of filter‐feeding fish and clams suppressed phytoplankton, which could not be affected by low‐level nutrients.
2. Bottom‐up effects at high‐level nutrients on phytoplankton overcome top‐down effects, indicating that nutrient levels should be controlled to optimize the effect of the intervention.

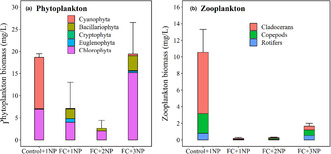

## 1 INTRODUCTION

Successful restoration of shallow eutrophic lakes is often marked by a shift from a turbid status dominated by phytoplankton to a clear water status dominated by submerged macrophytes (Jeppesen et al., 2005; Liu et al., 2018). However, there remains a competitive relationship between submerged macrophytes and phytoplankton (Lürig et al., 2021; Scheffer et al., 2001; Søndergaard et al., 2017). Submerged macrophytes can maintain a lake's clear water state by removing excess nutrients, inhibiting sediment resuspension, and reducing the biomass of phytoplankton (Han et al., 2022; Jeppesen et al., 2012; Lürig et al., 2021). Phytoplankton are prone to be limited by nutrients in the water column but have a clear competitive advantage in terms of light, while submerged macrophytes are able to obtain nutrients from the sediment, where the nutrient content is generally much higher than the water column, but are susceptible to light limitation (Chen et al., 2020; Han et al., 2022). Although submerged macrophytes are often the main primary producers in clear water lakes, the competitive advantage of phytoplankton increases with increased nutrient concentrations, resulting in lower water transparency that limits growth and even survival of submerged macrophytes, and which eventually might result in a shift back to a turbid water status (Jeppesen et al., 2012; Jin, van Leeuwen, Temmink, & Bakker, 2022; Liu et al., 2020; Zhang et al., 2022). Thus, a primary aim of biomanipulation in lake restoration is to improve light conditions for submerged macrophytes by reducing phytoplankton biomass and suspended sediments (Yu et al., 2016).

Phytoplankton biomass in freshwater ecosystems is determined by both top‐down effects of predators and bottom‐up effects of nutrients (He et al., 2021; Karpowicz et al., 2023). Traditional biomanipulation methods developed for northern temperate lakes attempt to enhance zooplankton grazing on phytoplankton by introducing piscivorous fish or by removing plankti‐benthivorous fish (Hilt et al., 2018; Jeppesen et al., 2012; Jin, van Leeuwen, Van de Waal, & Bakker, 2022). Benndorf et al. (2002) concluded that fish manipulation for top‐down control of phytoplankton was more likely to succeed in mesotrophic or slightly eutrophic lakes than in eutrophic or hypertrophic lakes. Jeppesen et al. (2012) argued that water column phosphorus concentrations should be kept less than 100 μg L^−1^, or the external phosphorus loads should be reduced to 0.5–2.0 g m^−2^ a^−1^ to achieve stability and successful biomanipulations. However, the higher primary productivity and more complex food webs found in lakes in tropical and subtropical regions is expected to further weaken the top‐down effect of traditional biomanipulations (Jeppesen et al., 2012). Liu et al. (2018) found strong bottom‐up but weak top‐down effects in a successful restoration of a tropical shallow eutrophic lake, indicating the importance of bottom‐up effects through reduced sediment resuspension and nutrient release, rather than the top‐down effects observed in many temperate lakes.

Lake Taihu is a typical subtropical large eutrophic shallow lake. Large‐scale basin‐wide management interventions were implemented following the 2007 water crisis (Zhou et al., 2021); however, 10 years of monitoring data (2008–2018) has shown that average annual external nutrient inputs (TN: 40–50,000 t; TP: 2000 t) have not been significantly reduced (Cheng et al., 2020; Qin, 2020). A previous study showed that the combined use of two filter‐feeding organisms with different feeding niches, that is, bighead carp and Asian clams, was effective in reducing the total phytoplankton biomass and improving water clarity (Shen et al., 2020). The efficacy of this approach was attributed to strong complementarity in terms of algae size that can be filtered, and the aggregation of small particles by bivalves, increasing the availability of food resource for filter‐feeding fish (de Rezende Ayroza et al., 2019; Shen et al., 2020). However, the stability of this biomanipulation intervention on nutrient loads and algal control is still unclear, especially when external nutrient inputs are not reduced (Parr et al., 2020).

Building on Shen et al. (2020) in this study, we focus on the interactions of filter‐feeding fish and bivalves and different nutrient concentrations to explore if nutrient thresholds exist for this form of biomanipulation. We hypothesized that when external nutrients exceed a certain threshold, bottom‐up effects of nutrients exceed top‐down control by predators, resulting in increased phytoplankton biomass. To test the hypothesis, an outdoor mesocosm experiment was conducted using different nutrient concentrations, and four treatments simulating daily loads of nutrients in Lake Taihu were studied: current average daily load (FC + 1NP), two times (FC + 2NP) and three times (FC + 3NP) average daily loads of nutrients with both fish and clams and as a control current daily loads without fish or bivalves.

## 2 MATERIALS AND METHODS

### 2.1 Experiment design

The outdoor mesocosm experiment was conducted from 12 September to 11 October 2018 at the Taihu Laboratory for Lake Ecosystem Research (TLLER), located at the northern edge of Lake Taihu. Lake Taihu (30°56′–31°34′ N, 119°54′–120°35′ E) is the third largest freshwater lake in China and a typical shallow eutrophic lake located in the south of the Yangtze River Delta. Lake Taihu has a surface area of 2338 km^2^, a mean depth of 1.9 m, and a mean water residence time of approximately 300 days.

Twelve polyethylene plastic tanks (82 cm height × 102 cm upper diameter × 85 cm bottom diameter) were filled with a 10 cm layer of prescreened (mesh size: 1.7 mm) sediment and 500 L filtered (mesh size: 64 μm) water collected from eutrophic Lake Taihu. A water pump (24 W) was fixed on the wall of each mesocosm 10 cm below the water surface to ensure water circulation with a run–stop ratio of 1 h:1 h. There was a 1‐week buffering and stabilization period before the beginning of the experiment.

To simulate the external nutrient loads in Lake Taihu, nitrogen (N) and phosphorus (P) were added daily to each mesocosm throughout the experiment. The mesocosm experiment comprised a total of four treatments: current average daily loads of N and P without filter‐feeding fish (F) or clams (C) (Control + 1NP) and three treatments at three nutrient concentrations with filter‐feeding fish and clams: current average daily load (FC + 1NP), two times (FC + 2NP), and three times (FC + 3NP) average daily loads (Table 1). Three replicates were run for each treatment. Nutrient concentrations used in the different treatments were based on average daily external nutrient loads of 130 μg N L^−1^ day^−1^ for nitrogen and 5 μg P L^−1^ day^−1^ for phosphorus, respectively (Paerl et al., 2011). N and P were added separately as aqueous solutions of potassium nitrate (KNO_3_) and potassium dihydrogen phosphate (KH_2_PO_4_), which are the main inorganic nutrients in Lake Taihu (Xu et al., 2021).

**TABLE 1:** Biomass of filter‐feeding fish and clams and N and P concentrations representing different daily nutrient loads in Lake Taihu: Control + 1NP = concentrations representing current average daily load and no fish or clams, and treatments with fish and clams (FC) at nutrient concentrations representing current (FC + 1NP), two times (FC + 2NP), and three times (FC + 3NP) average daily loads.

	Fish (g m^−3^)	Clam (g m^−2^)	Nitrogen (μg L^−1^ d^−1^)	Phosphorus (μg L^−1^ d^−1^)
Control + 1NP	0	0	130	5
FC + 1NP	80	200	130	5
FC + 2NP	80	200	260	10
FC + 3NP	80	200	390	15

The clams and bighead carp were collected from a nearby aquaculture pond and had an average weight of 3.15 ± 0.23 g/ind. and 10.41 ± 0.92 g/ind., respectively. Prior to the experiment, all clams and fish were kept in a lake‐side pond and the water was changed every 3 days. The biomass levels of fish and clams used here are based on optimal biomass having filter‐feeding effects on phytoplankton from a previous study (Shen et al., 2020). Each treatment with FC had 4 fish and 38 clams, and biomasses averaged 41.63 ± 1.34 g per tank for fish and 119.54 ± 0.90 g per tank for clams, respectively. During the experiment, fish were checked daily and no deaths were noted throughout the experiment. It was not possible to check clam densities during the experiment, however at the end of the experiment all the clams were collected and counted, with mean survival rates of 93.3 ± 3.0%. At the end of the experiment, the biomasses of fish and clams averaged 48.29 ± 4.00 g per tank and 120.97 ± 3.49 g per tank, respectively.

### 2.2 Sampling and analyses

Water samples (5 L) were taken every 5 days from two different depths (surface and 5 cm above the sediment) using a 2.5 L plexiglass water collector and mixed. The concentrations of total nitrogen (TN), total phosphorus (TP), total dissolved nitrogen (TDN), and total dissolved phosphorus (TDP) were determined through colorimetry after digestion with K_2_S_2_O_8_ and NaOH solution (Rice et al., 2017). Total suspended solid (TSS) was calculated from 100 to 200 mL water samples filtered through a pre‐combusted and pre‐weighted GF/C filters, which were then dried in a muffle furnace (MF1050, Jinlan Instrument Manufacturing Co., Ltd, Shanghai, China) to a constant weight at 105°C for 4 h. Chlorophyll *a* (Chl *a*) was determined on known amounts of GF/C filter water (Whatman International Ltd., Maidstone, England) using a spectrophotometer (UV‐2450, Shimadzu Co., Ltd., Japan) (Rice et al., 2017). Filters were kept frozen until analyzed and pigments were extracted using a 90% (v/v) acetone/water solution. Prior to collecting water samples, water temperature (WT), electrical conductivity (EC), pH, and dissolved oxygen (DO) were measured using a YSI 9500 photometer (YSI Inco, Yellow Springs, OH, USA).

Phytoplankton and zooplankton were sampled on Day 0 (before bighead carp and clams were put in) and Day 30 (the end) of the mesocosm experiment. A 1 L water sample was preserved in 1% Lugol's solution and after 48 h of sedimentation, the supernatant was removed, and the residue was collected for identifying and counting rotifers and phytoplankton using a 100×–400× microscope. Species were identified according to the taxonomic classification of Hu and Wei (2006) and Wang (1961) and their biomasses were calculated from cell number and cell size measurements assuming 1 mm^3^ volume equals 1 mg fresh weight biomass (Zhang & Huang, 1991). Crustacean zooplankton were collected by filtering 5 L of mixed water samples through a plankton net (64 μm mesh) and then preserved in 5% formaldehyde solution. Cladocerans and copepods were counted at 40× magnification and species identification was performed according to standard methods (Chiang & Du, 1979). Zooplankton biomass was estimated based on the weight‐body size regression of Huang (1999).

### 2.3 Statistical analyses

One‐way analysis of similarities (ANOSIM) was used to test for differences in phytoplankton and zooplankton community structure based on Bray–Curtis similarity of log(x + 1) transformed species biomass data. In addition, we calculated two food web metrics, that is, zooplankton to phytoplankton biomass ratio (Zoop:Phyt) and Chl *a* to TP ratio by weight (Chl *a*: TP), representing the top‐down effects of filter‐feeding fish and clams and bottom‐up effects of nutrients, respectively.

Filter‐feeding fish and clams (Control + 1NP and FC + 1NP) and nutrients (FC + 1NP, FC + 2NP and FC + 2NP) were set as fixed factors to explore the effects on water quality parameters, that is, Chl *a*, TP, TDP, TN, TDN, the TN to TP ratio by weight (N:P), TSS, WT, EC, pH and DO, and time was incorporated as a random effect in our generalized linear mixed models (GLMM) (Bolker et al., 2009). We also applied GLMM to explore the influence of filter‐feeding fish and clams and nutrients as fixed effects, respectively, on the biomass of phytoplankton and zooplankton, Zoop:Phyt and Chl *a*: TP at the end of the mesocosm experiment. We assumed Gaussian error distributions for all response variables and fitted modes using function *glmmTMB* from R package *glmmTMB* (Brooks et al., 2017). For each response variable, we assessed the relative fit of the two different candidate models (fixed effect: FC and null, or Nutrient and null, respectively) and selected the best model based on the lowest AIC value from the default ANOVA function.

## 3 RESULTS

### 3.1 Nutrients and physicochemical parameters in water column

**FIGURE 1:**
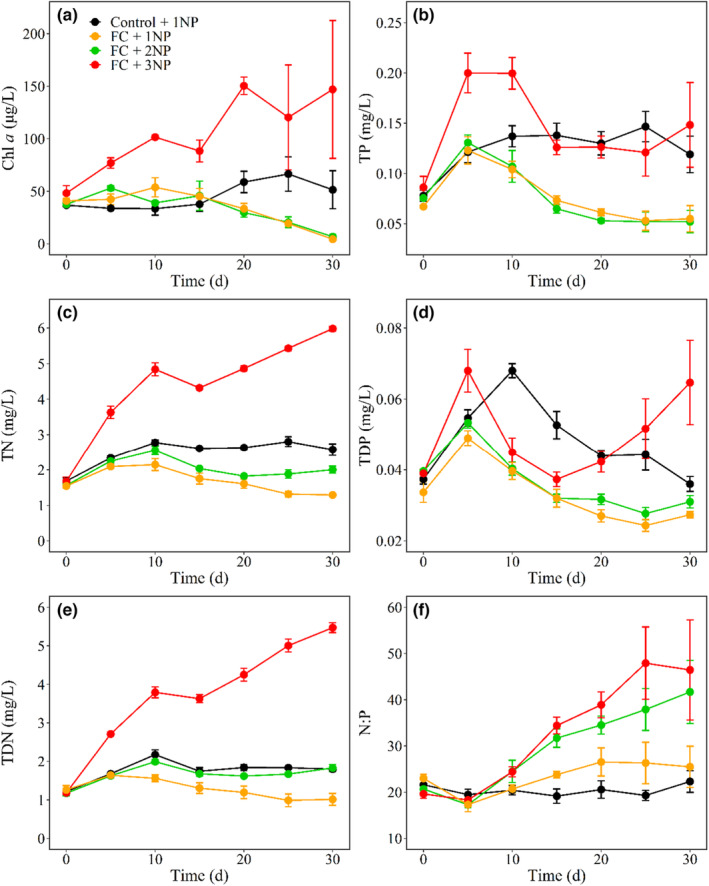
Time series plots of Chl *a*, TP, TN, TDP, TDN, and the TN to TP ratio by weight (N:P) in four treatments with nutrient concentrations representing current (1NP), two times (2NP), and three times (3NP) average daily loads of N and P in Lake Taihu and with (FC) or without (Control) filter‐feeding fish and clams.

**FIGURE 2:**
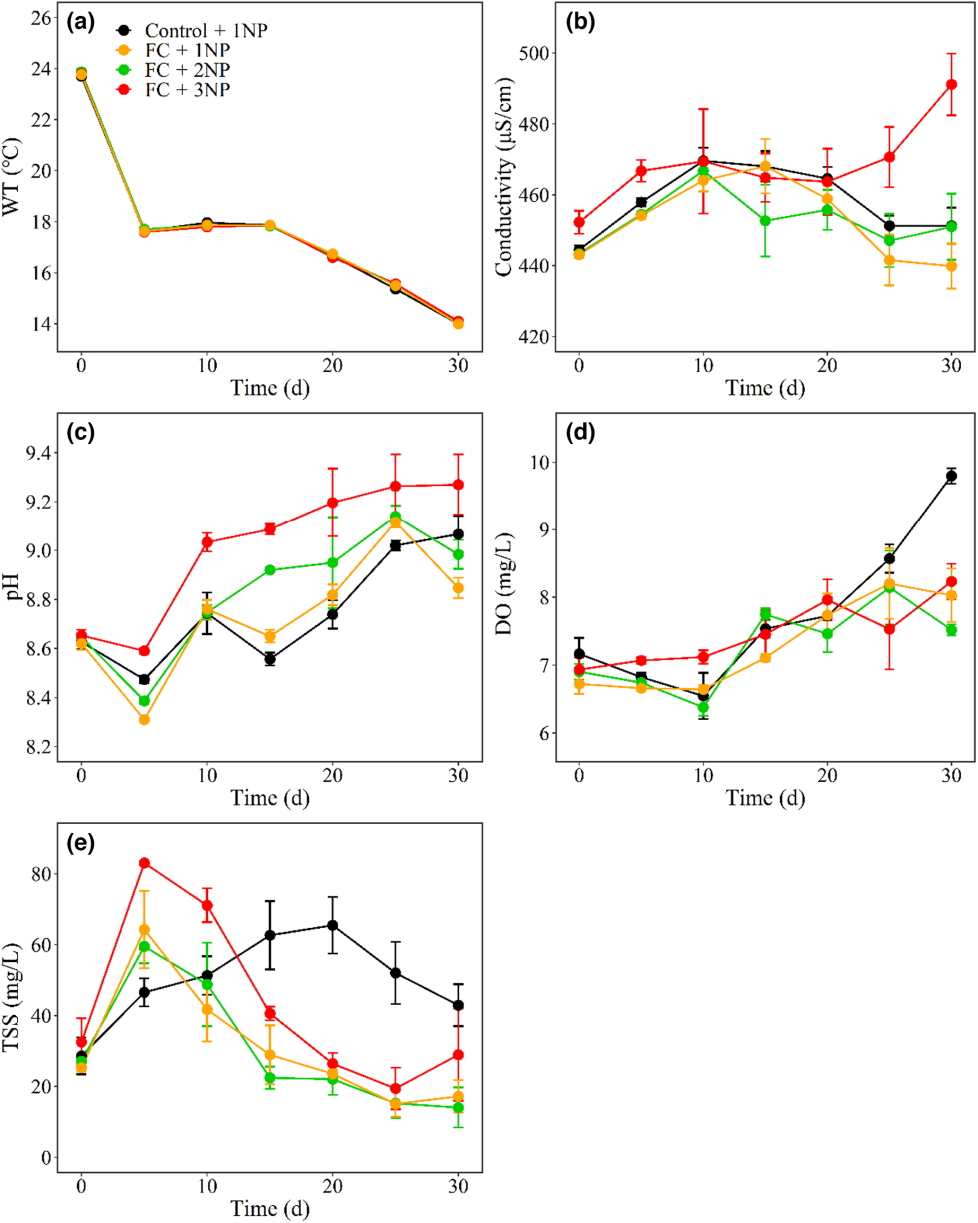
Time series plots of physicochemical parameters in four treatments with nutrient concentrations representing current (1NP), two times (2NP), and three times (3NP) average daily loads of N and P in Lake Taihu and with (FC) or without (Control) filter‐feeding fish and clams.

Negative effects of filter‐feeding fish and clams were found on TN, TP, TDN, TDP and TSS, and positive effects on N:P at nutrient concentrations representing current average daily nutrient loads (1NP) (Table 2, Figure 1). With the presence of filter‐feeding fish and clams, higher nutrient concentrations, positively affected N:P in both 2NP and 3NP treatments, while effects on TN, TP, TDN, TDP, and TSS were only evident in the 3NP treatment (Table 3, Figure 1). Neither filter‐feeding fish and clams nor nutrients had significant effects on WT or DO. No significant effects of filter‐feeding fish and clams were found on EC and pH, while higher nutrient concentrations positively affected EC (3NP) and pH (2NP and 3NP) (Table 3, Figure 2).

**TABLE 2:** GLMM results showing the effects of filter‐feeding fish and clams on Chl *a*, nutrients, water physicochemical parameters, phytoplankton, and zooplankton at nutrient concentrations representing current average daily loads of N and P.

Variables	Coefficients	Estimate	SE	z‐Value	*p*
Chl *a*	(Intercept)	45.55	4.03	11.30	**<.001**
	FC	−11.30	5.70	−1.98	**.047**
TN	(Intercept)	2.49	0.10	24.11	**<.001**
	FC	−0.81	0.09	−8.69	**<.001**
TP	(Intercept)	0.12	0.01	17.17	**<.001**
	FC	−0.05	0.01	−6.39	**<.001**
TDN	(Intercept)	1.76	0.08	21.97	**<.001**
	FC	−0.47	0.08	−6.33	**<.001**
TDP	(Intercept)	0.05	0.00	14.83	**<.001**
	FC	−0.01	0.00	−8.09	**<.001**
N:P	(Intercept)	20.42	0.89	22.87	**<.001**
	FC	2.89	1.18	2.45	**.014**
TSS	(Intercept)	49.92	4.32	11.57	**<.001**
	FC	−19.06	4.68	−4.07	**<.001**
WT	(Intercept)	17.61	1.07	16.44	**<.001**
EC	(Intercept)	455.49	3.62	125.80	**<.001**
pH	(Intercept)	8.74	0.08	109.90	**<.001**
DO	(Intercept)	7.52	0.31	24.63	**<.001**
Phytoplankton	(Intercept)	18.63	3.45	5.41	**<.001**
	FC	−11.54	4.87	−2.37	**.018**
Zooplankton	(Intercept)	10.55	1.59	6.61	**<.001**
	FC	−10.36	2.26	−4.59	**<.001**
Zoop:Phyto	(Intercept)	0.57	0.12	4.56	**<.001**
	FC	−0.37	0.18	−2.10	**.036**
Chl *a*:TP	(Intercept)	0.37	0.04	8.30	**<.001**
	FC	0.09	0.05	1.69	.091

*Note*: Significant terms in bold. All variables were modeled as a Gaussian distribution.

Abbreviations: Chl *a*:TP, chlorophyll *a* to total phosphorus; FC, filter‐feeding fish and clams; N:P, total nitrogen to total phosphorus; Zoop:Phyt, zooplankton to phytoplankton biomass.

**TABLE 3:** GLMM results showing the effects of filter‐feeding fish and clams on Chl *a*, nutrients, water physicochemical parameters, phytoplankton, and zooplankton at nutrient concentrations representing two times (2NP) and three times (3NP) average daily loads of N and P in Lake Taihu.

Variables	Coefficients	Estimate	SE	z‐Value	*p*
Chl *a*	(Intercept)	34.25	7.76	4.41	**<.001**
	2NP	−1.00	10.98	−0.09	.928
	3NP	70.47	10.98	6.42	**<.001**
TN	(Intercept)	1.68	0.22	7.61	**<.001**
	2NP	0.34	0.21	1.59	.111
	3NP	2.71	0.21	12.79	**<.001**
TP	(Intercept)	0.08	0.01	6.47	**<.001**
	2NP	0.00	0.01	−0.03	.980
	3NP	0.07	0.01	8.69	**<.001**
TDN	(Intercept)	1.28	0.22	5.90	**<.001**
	2NP	0.38	0.21	1.75	.080
	3NP	2.44	0.21	11.37	**<.001**
TDP	(Intercept)	0.03	0.00	10.37	**<.001**
	2NP	0.00	0.00	1.28	.201
	3NP	0.02	0.00	6.48	**<.001**
N:P	(Intercept)	23.31	3.10	7.51	**<.001**
	2NP	6.45	2.21	2.92	**.003**
	3NP	9.56	2.21	4.33	**<.001**
TSS	(Intercept)	30.87	7.17	4.30	**<.001**
	2NP	−0.97	4.05	−0.24	.811
	3NP	12.29	4.05	3.03	**.002**
WT	(Intercept)	17.62	1.07	16.44	**<.001**
EC	(Intercept)	452.82	3.26	138.86	**<.001**
	2NP	0.21	3.98	0.05	.957
	3NP	15.57	3.98	3.92	**<.001**
pH	(Intercept)	8.73	0.09	96.20	**<.001**
	2NP	0.09	0.04	2.43	**.015**
	3NP	0.28	0.04	7.35	**<.001**
DO	(Intercept)	7.35	0.19	38.59	**<.001**
Phytoplankton	(Intercept)	7.09	4.45	1.60	.111
	2NP	−4.46	6.29	−0.71	.478
	3NP	12.31	6.29	1.96	.050
Zooplankton	(Intercept)	0.19	0.18	1.05	.292
	2NP	0.09	0.25	0.37	.712
	3NP	1.47	0.25	5.83	**<.001**
Zoop:Phyto	(Intercept)	0.17	0.05	3.31	**.001**
Chl *a*:TP	(Intercept)	0.45	0.07	6.51	**<.001**
	2NP	−0.01	0.08	−0.13	.899
	3NP	0.29	0.08	3.54	**<.001**

*Note*: Significant terms in bold. All variables were modeled as a Gaussian distribution.

Abbreviations: Chl *a*:TP, chlorophyll *a* to total phosphorus; N:P, total nitrogen to total phosphorus; Zoop:Phyt, zooplankton to phytoplankton biomass.

### 3.2 Chlorophyll *a* concentrations

Filter‐feeding fish and clams negatively affected Chl *a* concentration at nutrient concentrations representing current normal daily nutrient loads (1NP) (Table 2, Figure 1). With the presence of filter‐feeding fish and clams, effects of higher nutrient concentrations on Chl *a* concentration were only evident in the 3NP treatment, but not in the 2NP treatment (Table 3, Figure 1). Specifically, the concentrations of Chl *a* at the beginning of the experiment ranged from between 37.6 and 45.6 μg L^−1^, with no differences among treatments. At the end of the experiment, the mean Chl *a* concentration in Control +1NP was 51.51 ± 18.09 μg L^−1^, that is, 11x higher than the FC + 1NP (4.50 ± 1.13 μg L^−1^) treatment. Moreover, mean Chl *a* concentration in the FC + 3NP treatment was 146.99 ± 65.60 μg L^−1^, 33× and 22× higher than the FC + 1NP and FC + 2NP (6.83 ± 2.13 μg L^−1^) treatments, respectively (Figure 1).

### 3.3 Phytoplankton biomass and community composition

Phytoplankton abundance and biomass at the start of the mesocosm experiment averaged 4.5 ± 1.5 × 10^6^ ind. L^−1^ and 1.8 ± 0.5 mg L^−1^, respectively, and did not differ among treatments. Cyanophyta and Chlorophyta contributed to >90% of the phytoplankton abundance, with *Microcystis* sp. (Cyanophyta) and *Scenedesmus* sp. (Chlorophyta) predominating, while *Scenedesmus* sp. (Chlorophyta) and *Euglena* sp. (Euglenophyta) predominated in terms of biomass.

**FIGURE 3:**
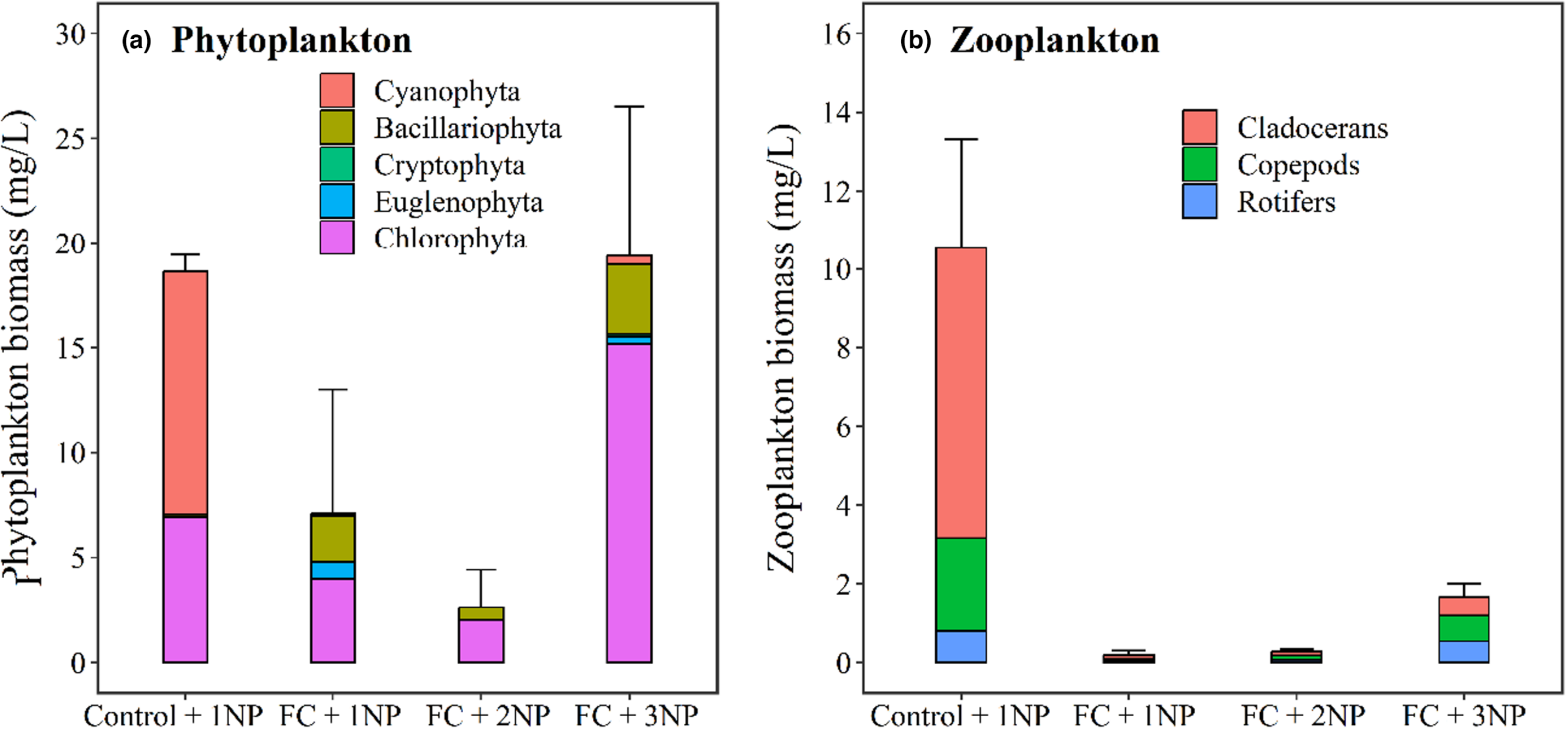
Phytoplankton (a) and zooplankton (b) biomass at the end of the experiment. Four treatments with nutrient concentrations representing current (1NP), two times (2NP), and three times (3NP) average daily loads of N and P in Lake Taihu and with (FC) or without (control) filter‐feeding fish and clams.

At the end of the experiment, phytoplankton biomass was lower in the FC + 1NP and FC + 2NP treatments and higher in the FC + 3NP treatment (Tables 2, 3, Figure 3). Although total phytoplankton biomass did not differ in the Control + 1NP and FC + 3NP treatments (*p* > .05), phytoplankton composition did (*p* < .05). The Control + 1NP treatment was dominated by Cyanophyta (mainly *Microcystis* sp.), accounting for 61.8% of the total biomass and Chlorophyta (37.4%), while the FC + 3NP treatment was dominated by Chlorophyta (mainly *Scenedesmus dimorphus* and *Pediastrum duplex*), accounting for 73.5% of the total biomass and Bacillariophyta (18.6%). The FC + 1NP and FC + 2NP treatments had similar phytoplankton assemblages and were mainly dominated by Chlorophyta and Bacillariophyta (Figure 3).

### 3.4 Zooplankton biomass and community

Zooplankton biomass at the beginning of the mesocosm experiment averaged 1.8 ± 0.5 mg L^−1^ and did not differ among treatments. Cladocerans dominated in all mesocosms (reaching 84.7 ± 27.3%), with *Bosmina* sp. and *Ceriodaphnia cornuta* as the predominant species.

At the end of the mesocosm experiment, cladocerans still dominated in the Control + 1NP treatment. Zooplankton biomass in FC + 1NP treatment decreased significantly compared with Control + 1NP treatment, while the zooplankton biomass in FC + 3NP treatment increased significantly compared with FC + 1NP and FC + 2NP (Figure 3). Rotifers, mainly *Keratella* sp. and *Brachionus* sp., had the highest biomass in the FC + 3NP treatment (41.4%), followed by branchiopods (33.2%) and copepods (25.4%) at the end of the mesocosm experiment (Figure 3).

### 3.5 Top‐down and bottom‐up effects

**FIGURE 4:**
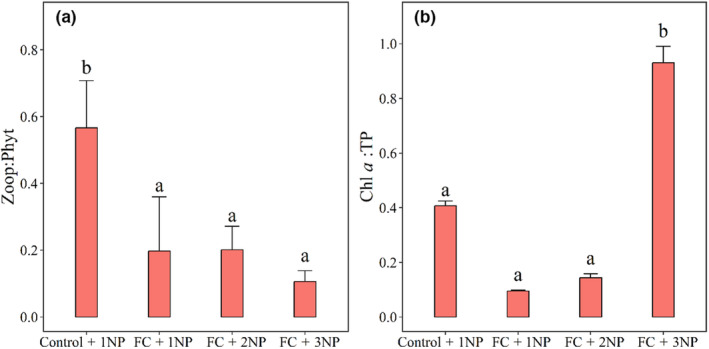
Top‐down effects of filter‐feeding fish and clams and bottom‐up effects of nutrients on zooplankton to phytoplankton biomass (Zoop:Phyt; a) and Chl *a* to TP (Chl *a*: TP; b) at the end of the experiment. Four treatments with nutrient concentrations representing current (1NP), two times (2NP) and three times (3NP) average daily loads of N and P in Lake Taihu and with (FC) or without (control) filter‐feeding fish and clams. Values represented mean ± SE (*n* = 3). Different letters show significant differences (*p* < .05).

Zoop:Phyt was lower in all treatments with filter‐feed fish and clams, and GLMM revealed no significant effects of nutrients (Tables 2, 3, Figure 4). However, at high nutrient concentrations (3NP) we found significantly positive effects on Chl *a*:TP, while effects of filter‐feeding fish and clams on Chl *a*:TP were not significant at current average daily nutrient loads (1NP) (Tables 2, 3, Figure 4).

## 4 DISCUSSION

Consistent with our hypothesis, we found that when nutrient concentrations exceeded twice the current average daily loads of Lake Taihu, the bottom‐up effect of nutrients overrode the top‐down control of predators, resulting in an increase in phytoplankton biomass. This finding indicates that the top‐down effects of filter‐feeding fish and bivalves on phytoplankton can moderate the bottom‐up effects of high nutrient loads.

The findings that the combination of filter‐feeding fish and clams resulted in lower water column biomass of phytoplankton, as well as the concentrations of nutrients, TSS and Chl *a*, are consistent with our previous work (Shen et al., 2020). Furthermore, these results indicated that the combination of filter‐feeding fish and clams could effectively control the phytoplankton biomass at current average daily nutrient loads in Lake Taihu, but not at three times daily nutrient loads. Although zooplankton biomass (*Bosmina* sp.) increased significantly in the control treatment without fish and clams, total phytoplankton biomass and the proportion of cyanobacteria also increased. These results could be explained by the volume‐efficiency hypothesis proposed by Brooks and Dodson (1965). For example, while *Bosmina* sp. can feed on particle sizes of 70–80 μm, they are unable to feed on cyanobacterial colonies, most of which were larger than 80 μm (Burns, 1968; Xiao et al., 2018). Hence, feeding on smaller algae might contribute to cyanobacterial blooms by reducing competition with other algae. In contrast, filter‐feeding fish and clams have different filter‐feeding preferences, with bighead carp feeding more efficiently on large‐celled or colonies of algae while clams prefer smaller algae (Shen et al., 2020; Wang et al., 2018). Therefore, their combination and interactions extend the top‐down control for phytoplankton.

High external nutrient loadings could also significantly affect the efficacy of biomanipulations when using filter‐feeding fish and clams (Karpowicz et al., 2023). In our treatments with filter‐feeding fish and clams, Chl *a* concentrations and phytoplankton biomass were highest in nutrient concentrations representing three times average daily nutrient loads, but no differences were found at lower nutrient concentrations (FC + 1NP and FC + 2NP). The increase in phytoplankton biomass in the FC + 3NP treatment may be due to excessive nutrients that negated top‐down control of filter‐feeding fish and clams resulting in an increase in Chl *a* and phytoplankton biomass (Mao et al., 2020; Rettig & Smith, 2021; Shen et al., 2021). Although *Microcystis aeruginosa* blooms occurred in the control treatment, Chlorophyta predominated at the highest nutrient concentrations (3NP), a finding that might be explained by N:P in the water column (Chen et al., 2018; He et al., 2021; Shatwell & Köhler, 2019). Smith (1983) concluded from an analysis of growing season data from 17 lakes throughout the world that Cyanophyta tend to dominate at N:P < 29 while the relative proportion of Cyanophyta decreases at N:P > 29. This was consistent with our results that the N:P at the highest nutrient concentration (3NP) was much greater than 29, while that in the no‐fish‐clam treatment (Control + 1NP) N:P was close to this value.

It is well known that filter‐feeding fish are able to strongly suppress zooplankton biomass, especially larger sized, for example, cladocerans (Yin et al., 2022) and that bivalves can compete with zooplankton for food resources resulting in reduced zooplankton biomass (Atkinson et al., 2021; Rojas Molina et al., 2012). This assumption was also supported by our mesocosm results. Zooplankton biomass in the treatments with filter feeders (FC + 1NP and FC + 2NP) were at very low at the end of the experiment and much higher in FC + 3NP treatment. Furthermore, the highest nutrient concentrations (3NP) had a positive effect on zooplankton and phytoplankton biomass, while no significant effects were noted at the two lower nutrient concentrations with filter‐feeding fish and clams. This is mainly because the highest nutrient concentration studied here supported higher growth of phytoplankton, providing food for small‐sized rotifers, and predation by fish was likely weaker than that of cladocerans and copepods (Mao et al., 2020). Moreover, it is plausible that the high phytoplankton biomass in the FC + 3NP treatment resulted in lower water transparency, which may also have hindered fish predation on zooplankton (Chen et al., 2021; He et al., 2020).

## 5 CONCLUSION

Our results indicated that when external nutrient loads were twice the current average daily external loads of Taihu Lake (TN ≤ 260 μg L^−1^ d^−1^; TP ≤ 10 μg L^−1^ d^−1^), the combined stocking of filter‐feeding fish and bivalves (80 g m^−3^ bighead carp; 200 g cm^−2^ clams) could effectively control water column Chl *a* concentrations and phytoplankton biomass. However, at higher nutrient loads the top‐down control of phytoplankton by filter‐feeding fish and bivalves might not be as effective, resulting in an increase in the bottom‐up effects and increased of phytoplankton biomass. Thus, in order to ensure the efficacy of biomanipulation interventions using filter‐feeding fish and bivalves for algal control efforts should focus on controlling nutrient loads.

## AUTHOR CONTRIBUTIONS

**You Zhang:** Conceptualization (equal); data curation (lead); formal analysis (lead); funding acquisition (equal); methodology (equal); software (lead); writing – original draft (lead). **Ruijie Shen:** Conceptualization (equal); data curation (equal); formal analysis (equal); methodology (equal); software (equal); writing – original draft (supporting). **Kuanyi Li:** Conceptualization (equal); funding acquisition (equal); writing – review and editing (equal). **Qisheng Li:** Methodology (equal). **Huihui Chen:** Methodology (equal). **Hu He:** Data curation (equal); formal analysis (supporting). **Xiaohong Gu:** Funding acquisition (equal); writing – review and editing (equal). **Zhigang Mao:** Conceptualization (equal); writing – original draft (supporting). **Richard K. Johnson:** Writing – review and editing (equal).

## CONFLICT OF INTEREST STATEMENT

We declare there is no conflict of interest associated with any of the decisions made or components of this study.

## Supporting information


Data S1.

## Data Availability

All data are provided as Supporting Information.
